# Ternary All-Polymer Solar Cells With 8.5% Power Conversion Efficiency and Excellent Thermal Stability

**DOI:** 10.3389/fchem.2020.00302

**Published:** 2020-04-21

**Authors:** Xi Liu, Chaohong Zhang, Shuting Pang, Ning Li, Christoph J. Brabec, Chunhui Duan, Fei Huang, Yong Cao

**Affiliations:** ^1^School of Textile Materials and Engineering, Wuyi University, Jiangmen, China; ^2^State Key Laboratory of Luminescent Materials and Devices, Institute of Polymer Optoelectronic Materials and Devices, South China University of Technology, Guangzhou, China; ^3^Institute of Materials for Electronics and Energy Technology (i-MEET), Friedrich-Alexander-Universität Erlangen-Nürnberg, Erlangen, Germany; ^4^SUSTech Academy for Advanced Interdisciplinary Studies, Southern University of Science and Technology, Shenzhen, China; ^5^Helmholtz Institute Erlangen-Nürnberg for Renewable Energy (HI ERN), Erlangen, Germany

**Keywords:** all-polymer solar cells, ternary solar cells, power conversion efficiency, thermal stability, Förster resonant energy transfer

## Abstract

All-polymer solar cells (all-PSCs) composed of polymer donors and acceptors have attracted widespread attention in recent years. However, the broad and efficient photon utilization of polymer:polymer blend films remains challenging. In our previous work, we developed NOE10, a linear oligoethylene oxide (OE) side-chain modified naphthalene diimide (NDI)-based polymer acceptor which exhibited a power conversion efficiency (PCE) of 8.1% when blended with a wide-bandgap polymer donor PBDT-TAZ. Herein, we report a ternary all-PSC strategy of incorporating a state-of-the-art narrow bandgap polymer (PTB7-Th) into the PBDT-TAZ:NOE10 binary system, which enables 8.5% PCEs within a broad ternary polymer ratio. We further demonstrate that, compared to the binary system, the improved photovoltaic performance of ternary all-PSCs benefits from the combined effect of enhanced photon absorption, more efficient charge generation, and balanced charge transport. Meanwhile, similar to the binary system, the ternary all-PSC also shows excellent thermal stability, maintaining 98% initial PCE after aging for 300 h at 65°C. This work demonstrates that the introduction of a narrow-bandgap polymer as a third photoactive component into ternary all-PSCs is an effective strategy to realize highly efficient and stable all-PSCs.

## Introduction

Bulk-heterojunction (BHJ) polymer solar cells (PSCs) are a promising solar-energy technology due to their low cost, easy fabrication, light weight, and mechanical flexibility (Yu et al., 1995; Thompson and Fréchet, 2008; Brabec et al., 2010; Andersen et al., 2014; Lu et al., 2015b; Huang et al., 2019). In recent years, PSCs have achieved power conversion efficiencies (PCEs) of over 16% via the development of novel photoactive materials, optimized morphological control, and improved interface and device engineering (Meng et al., 2018; An Q. et al., 2019; Chang et al., 2019; Fan et al., 2019a; Li K. et al., 2019; Yan et al., 2019; Yuan et al., 2019; Yu et al., 2019). Specifically, one important effort has been the creation of novel photoactive acceptors beyond fullerene-based acceptors, aiming to mitigate the drawbacks of fullerene-based materials such as their expensive synthetic cost, weak optical absorption, finited bandgap, and morphological instability (Hummelen et al., 1995; Wienk et al., 2003; Cheng and Zhan, 2016). Therefore, there is increasing interest in developing and comprehending non-fullerene acceptors (Brabec et al., 2010; Nielsen et al., 2015; Kang et al., 2016; Cheng et al., 2018; Hou et al., 2018; Liu et al., 2018a; Yan et al., 2018; Yang et al., 2019b). Among these non-fullerene acceptors, polymeric electron acceptors were reported to have tunable absorption, modulable energy levels, and stable BHJ morphology (Li et al., 2014; Jung et al., 2015, 2016; Dou et al., 2016; Kang et al., 2016; Wang et al., 2017; Liu et al., 2018b; An N. et al., 2019; Yang et al., 2019a). Thus, all-polymer solar cells (all-PSCs) consisting of a polymeric donor and acceptor have attracted more and more attention and are promising for use in realizing highly efficient and stable solar cells (Kim et al., 2015; Kang et al., 2016; Liu et al., 2016, 2018b; Long et al., 2016; Wang et al., 2017; Zhang et al., 2017). Encouragingly, all-PSCs have recently achieved over 10% PCEs (Fan et al., 2017a,b, 2018, 2019b; Li et al., 2017a; Zhang et al., 2017; Chen et al., 2018; Kolhe et al., 2019; Li Z. et al., 2019; Meng et al., 2019; Yao et al., 2019; Zhu et al., 2019; Zhao et al., 2020). To date, highly efficient all-PSCs are mostly based on naphthalene diimide (NDI) polymer acceptors, because of their high electron mobility, suitable energy levels, and tunable BHJ morphology (Gao et al., 2016; Li et al., 2016; Fan et al., 2017a,b; Liu et al., 2018b). For example, poly[[*N, N*′-bis(2-octyldodecyl)-naphthalene-1,4,5,8-bis(dicarboximide)-2,6-diyl]-*alt*-5,5′-(2,2′-bithiophene)], is a state-of-the-art polymer acceptor with the commercial name N2200 (Yan et al., 2009; Fan et al., 2017b). However, the relatively weak absorption coefficient in near-infrared wavelengths of N2200 or its analogs prevents devices from attaining higher photocurrent responses and short-circuit current densities (*J*_sc_) (Fan et al., 2017a,b; Liu et al., 2018b). State-of-the-art all-PSCs usually exhibit lower than 40% external quantum efficiencies (EQEs) in the 700–800 nm wavelength range, which offered by N2200, seriously limiting further improvement of their *J*_sc_ and PCE values (Fan et al., 2017a,b; Liu et al., 2018b).

Ternary all-PSCs are based on the incorporation of a third polymer component into a binary polymer: polymer blend, thereby effectively improving device efficiencies via extending and/or enhancing light absorption, manipulating energy levels, and regulating active layer morphology (Huang et al., 2017; Fu et al., 2018; Xu and Gao, 2018; Yu et al., 2018; Gasparini et al., 2019; Lee et al., 2019). Jenekhe et al. developed a ternary all-PSC with a PCE of 3.2% composed of a polymer donor and two polymer acceptors (Hwang et al., 2015). Using the same ternary approach, Ito et al. (Benten et al., 2016), Li et al. (Su et al., 2016), and Wang et al. (Li et al., 2017a) constructed efficient ternary all-PSCs by combining wide-bandgap polymers (PCDTBT, PBDD-ff4T, and PBDTTS-FTAZ, respectively), with the narrow-bandgap PTB7-Th:N2200 blend, where the wide-bandgap polymers contributed to complementary absorption and improved photocurrent, resulting in steadily increased PCEs of 6.7, 7.2, and 9.0%, respectively. Recently, Ying et al. realized several ternary all-PSCs, which achieved PCEs over 10%; the high efficiencies of these ternary all-PSCs were attributed to the complementary absorption, enhanced photo-harvesting, improved charge-carrier transportation, and inhibited recombination (Fan et al., 2018, 2019b; Li Z. et al., 2019).

Considering the future practical applications of PSCs, device stability is a significant issue beyond its contribution to high photovoltaic efficiency. Specifically, device stability issues include the oxidation of electrodes, degradation of interface layers, and intrinsic instability of photoactive layer morphology under light and thermal aging (Jørgensen et al., 2012; Cheng and Zhan, 2016; Holliday et al., 2016; Baran et al., 2017; Kim et al., 2017; Mateker and McGehee, 2017; Zhang et al., 2018b; Hu et al., 2019; Speller et al., 2019). The ternary strategy has displayed potential as a useful approach for achieving stable solar cells (Kim et al., 2017; Zhang et al., 2019b). For example, the research groups of McCulloch (Baran et al., 2017), Kim (Kim et al., 2017), and Ade (Hu et al., 2019) all demonstrated small molecule acceptor-based ternary systems with excellent thermal stabilities, mainly due to controlled crystallization and miscibility achieved through the incorporation of a third component. Moreover, we and others have demonstrated the excellent long-term and thermal stabilities of binary or tandem all-PSCs through effective material design and device engineering (Li et al., 2017b; Liu et al., 2018b; Zhang et al., 2018a,c). However, ternary all-PSCs with high efficiencies and excellent thermal stabilities have not been widely investigated (Li et al., 2017a).

Previously, we have reported a linear oligoethylene oxide (OE) side-chain modified NDI-based polymer acceptor (NOE10) which offered a high efficiency (PCE of 8.1%) and excellent long-term stability when blended with a wide-bandgap polymer donor (PBDT-TAZ) to form binary all-PSCs (Liu et al., 2018b). In this work, we further improved the efficiency of all-PSCs through the ternary strategy while maintaining excellent thermal stability beyond that of binary all-PSCs. Specifically, the ternary all-PSCs were constructed by combining a state-of-the-art narrow-bandgap polymer (PTB7-Th) into the PBDT-TAZ:NOE10 binary blend. The ternary all-PSCs enable a PCE of 8.5% within a broad ternary polymer ratio, representing an 18% improvement over the corresponding binary all-PSCs. The enhanced device performance of the ternary all-PSCs stem from the combined effects of improved photon absorption, the generation of more free charges through simultaneous charge and energy transfer, and balanced charge transport. More importantly, the ternary all-PSCs exhibit excellent thermal stability, maintaining 98% of their initial PCE after aging for 300 h at 65°C. This work demonstrates that the introduction of the state-of-the-art narrow-bandgap polymer PTB7-Th as a third photoactive component positions ternary PBDT-TAZ:PTB7-Th:NOE10 all-PSCs as highly efficient and stable all-PSCs. Further, the high performances of ternary all-PSCs within broad ternary polymer ratios offer benefits for future large-scale technological applications.

## Results and Discussion

### Polymer Selection and Characterization

**Figure 1:**
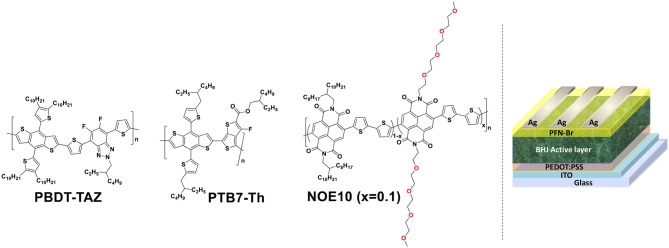
Chemical structures of photoactive layer polymers (PBDT-TAZ, PTB7-Th, and NOE10) and the device architecture schematic diagram.

The acceptor polymer NOE10 is a linear oligoethylene oxide (OE) side-chain modified naphthalene diimide (NDI)-based polymer reported by our group previously and presented in Figure 1. It can achieve a high PCE of ≈8% when used in a PBDT-TAZ:NOE10-based all-PSC due to its optimal photoactive layer morphology (Liu et al., 2018b). The donor polymer PBDT-TAZ is a wide-bandgap conjugated polymer derived from a benzodithiophene (BDT) building block and a difluorobenzotriazole (TAZ) unit with a bandgap >1.9 eV; PBDT-TAZ and its analogs have been widely applied in efficient all-PSCs demonstrated by our group and others (Li et al., 2016, 2017a; Duan et al., 2018; Liu et al., 2018b; Pang et al., 2019). Though the PBDT-TAZ:NOE10-based binary all-PSC showed a high PCE of 8.1% in our previous work, the binary blend shows weak absorption in the range of 600–800 nm, which restricts the further improvement of its quantum efficiency, *J*_sc_, and PCE. The state-of-the-art narrow-bandgap polymer PTB7-Th offers high absorption coefficient in the 600–800 nm range, and is a reasonable candidate for application as the third component in a binary PBDT-TAZ:NOE10 blend to improve long-wavelength absorption. Thus, PBDT-TAZ:PTB7-Th:NOE10 blends with different ratios were applied as the photoactive layer in a ternary all-PSCs system. The ratio of PBDT-TAZ:NOE10 was fixed at 1.5:1, while the PTB7-Th content was varied to optimize the polymer ratios.

**Figure 2:**
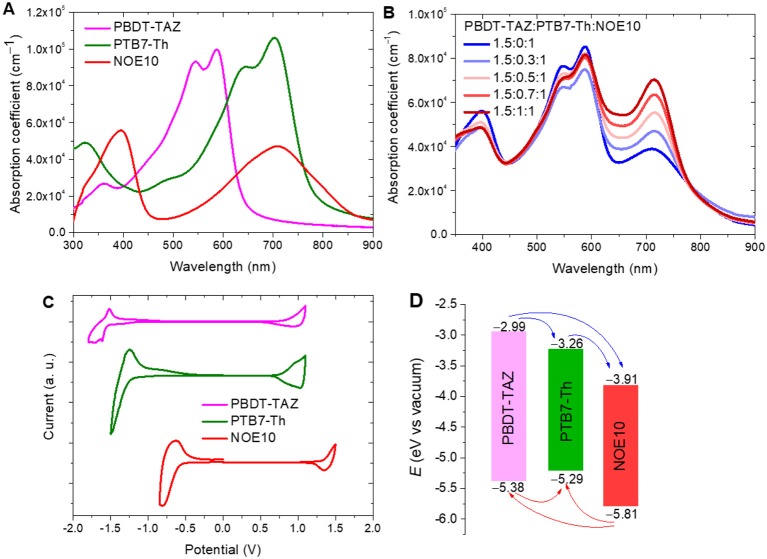
**(A)** The absorption spectra of the neat polymers films (PBDT-TAZ, PTB7-Th, and NOE10); **(B)** absorption spectra of the corresponding blend films; **(C)** cyclic voltammetry curves of PBDT-TAZ, PTB7-Th, and NOE10; **(D)** calculated energy level diagram of the polymers.

The optical absorption spectra of the photoactive layer polymers (PBDT-TAZ, PTB7-Th, and NOE10) and the corresponding blended films were shown in Figure 2A. The wide-bandgap donor polymer PBDT-TAZ exhibits absorption coefficients over 8 × 10^4^ cm^−1^ in the 500–600 nm range, whereas acceptor polymer NOE10 shows relatively weak absorption coefficients in the 600–850 nm range, which limits the light-harvesting efficiency of the PBDT-TAZ:NOE10 binary blend. The narrow-bandgap polymer PTB7-Th shows high absorption coefficient in the range of 600–760 nm with a maximal absorption coefficient of 1.05 × 10^5^ cm^−1^ at 705 nm, which complements the absorption of the binary system. The absorption spectra of the ternary blends representing different polymer ratios are shown in Figure 2B. As PTB7-Th content increases, the corresponding ternary blends clearly exhibit significantly enhanced absorption coefficient in the range of 650–760 nm. These results demonstrate that the introduction of PTB7-Th could improve the absorption of the ternary blend.

The electrochemical characteristic curves of the photoactive layer polymers were recorded using cyclic voltammetry (CV). Relevant CV curves are depicted in Figure 2C, and the calculated energy diagrams are shown in Figure 2D. The lowest unoccupied molecular orbital (LUMO) levels of PBDT-TAZ, PTB7-Th, and NOE10 are −2.99, −3.26, and −3.91 eV, respectively, which provide a cascading alignment for electron transfer. Meanwhile, the highest occupied molecular orbital (HOMO) levels of PBDT-TAZ, PTB7-Th, and NOE10 are −5.38, −5.29, and −5.81 eV, respectively, which indicates that the HOMO and LUMO levels of PTB7-Th fall between the HOMO and LUMO levels of PBDT-TAZ. The slightly increased HOMO level of PTB7-Th suggests that the partial holes generated from PBDT-TAZ may ultimately be transferred to the HOMO of PTB7-Th before extraction.

### Photovoltaic Properties

**Figure 3:**
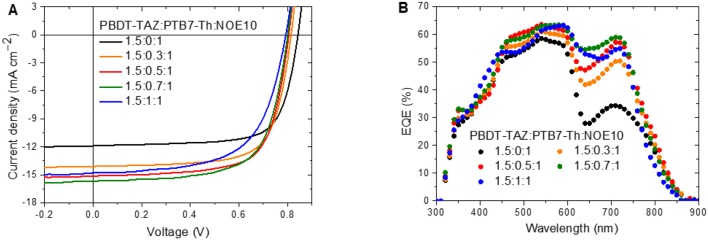
**(A)** *J*–*V* curves of the all-PSC devices; **(B)** EQE curves of the corresponding devices.

BHJ all-PSCs based on the photoactive layer polymers PBDT-TAZ, PTB7-Th, and NOE10 were fabricated with a device structure of ITO/PEDOT:PSS/photoactive layer/PFN-Br/Ag. As shown in Table 1, Figure S1 and Table S1, the ternary blend was optimized in terms of its detailed ternary ratio to maximize the PCE. The optimal ternary blended film was fabricated with a PBDT-TAZ:PTB7-Th:NOE10 weight ratio of 1.5:x:1 (x was set to be 0.1–1). Current density–voltage (*J*–*V*) characteristic curves and EQE curves of the champion devices using each ternary blend are exhibited in Figures 3A,B, and the corresponding photovoltaic parameters are listed in Table 1. There are a few notable results. First, the open-circuit voltage (*V*_oc_) gradually decreased as the PTB7-Th content increased from 0 to 100%. The linear dependence of *V*_oc_ on the loading of PTB7-Th indicates that the partial holes generated from PBDT-TAZ may ultimately be transferred to the HOMO of PTB7-Th before extraction. Wang et al. presented a similar ternary system but reported a nearly constant *V*_oc_, which may be attributable to the high weight ratio of PTB7-Th in the corresponding ternary blends (Li et al., 2017a). Second, there was a steady increase in *J*_sc_ as the PTB7-Th content increased from 0 to 80%, and forming a *J*_sc_ platform at 60–80% of PTB7-Th containing. These trends are consistent with absorption and EQE curves of the corresponding blends. The decreased *J*_sc_ of the 1.5:1:1 ternary ratio-based device should be due to the blend's unbalanced charge transport, which is discussed in the following sections. Third, the fill factor (FF) of the corresponding device decreased slightly with an increase of PTB7-Th, meanwhile, FF remains at a high level above 0.70 in 1.5:0:1–1.5:0.5:1 ternary ratio-based device. Our results indicate that the overall PCE can exceed 8% under a wide range of ternary ratios from 1.5:0.3:1 to 1.5:0.8:1; moreover, ternary all-PSCs with efficiency of 8.5% can be achieved with ternary ratios ranging from 1.5:0.5:1 to 1.5:0.7:1. Thus, these results suggest that the PBDT-TAZ:PTB7-Th:NOE10 ternary blend is a promising photoactive layer for use in high efficiency all-PSCs; furthermore, such ternary polymer-blend systems do not require precisely controlled ternary ratios, which increases the potential of ternary all-PSCs for use in large-scale commercial applications.

**Table 1:** The detail photovoltaic properties of the devices.

**D1:D2:A^a^**	***V*_**oc**_ [V]**	***J*_**sc**_ [mA cm^**−2**^]**	**FF**	**PCE [%]^b^**
1.5:0:1	0.843	11.9	0.72	7.2 (7.0 ± 0.2)
1.5:0.2:1	0.821	13.1	0.73	7.8 (7.6 ± 0.2)
1.5:0.3:1	0.814	14.1	0.71	8.2 (8.1 ± 0.1)
1.5:0.4:1	0.810	14.2	0.70	8.1 (8.0 ± 0.2)
1.5:0.5:1	0.805	15.1	0.70	8.5 (8.5 ± 0.1)
1.5:0.6:1	0.803	15.5	0.68	8.4 (8.3 ± 0.2)
1.5:0.7:1	0.801	15.7	0.68	8.5 (8.5 ± 0.1)
1.5:0.8:1	0.796	15.6	0.66	8.2 (8.0 ± 0.2)
1.5:1:1	0.792	14.8	0.62	7.3 (7.2 ± 0.1)

a D1 (PBDT-TAZ), D2 (PTB7-Th), A (NOE10);

b *the average values and standard deviations of statistics from the eight devices are given in parentheses*.

The EQE curves of the ternary all-PSCs are exhibited in Figure 3B. EQE responses of the corresponding ternary all-PSCs combined with absorption of the blends reflect the impact of the photoactive layer polymers on *J*_sc_. Compared to all-PSCs with a ternary ratio of 1.5:0:1, the EQE response of ternary all-PSCs including PTB7-Th is significantly improved within the range of 450 to 780 nm. In particular, the all-PSCs with ternary ratios of 1.5:0.5:1, 1.5:0.7:1, and 1.5:1:1 exhibit ≈60% EQE values at 450–750 nm. The specific EQE response indicates that the PBDT-TAZ:PTB7-Th:NOE10 ternary blend offers efficient electron and hole transfer.

### Charge Generation, Transport, and Recombination

**Figure 4:**
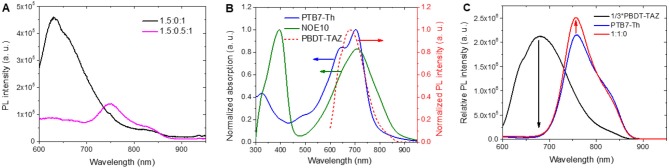
**(A)** The films PL curves excited at 500 nm; **(B)** absorption spectra of pure PTB7-Th and NOE10 films, and PL curve of pure PBDT-TAZ film excited at 500 nm; **(C)** PL spectra of pure PBDT-TAZ, pure PTB7-Th, and PBDT-TAZ:PTB7-Th blended film excited at 500 nm.

Photoluminescence (PL) tests were conducted to analyze exciton dissociation efficiency and the energy transfer mechanism in blended films. As shown in Figure 4A, the PBDT-TAZ:NOE10 binary blend (1.5:0:1) shows a PL peak at 625 nm; however the PL is completely quenched in the 600–700 nm range of the 1.5:0.5:1 ternary blend, and the slight PL signals at 700–850 nm contributed from the incomplete quenching of PTB7-Th (Figure S2A). This suggests that the incorporation of PTB7-Th improves exciton dissociation in the 1.5:0.5:1 ternary blend as compared to that in the 1.5:0:1 binary blend. Furthermore, to explain the more efficient exciton dissociation process in the ternary blends, we investigated the energy transfer mechanisms in the blended films with their corresponding PL spectra. As exhibited in Figure 4B, the PL spectrum of PBDT-TAZ strongly overlaps with the absorption range of PTB7-Th and NOE10, providing sufficient spectral overlap between the emission of the energy donor (PBDT-TAZ) and the absorption of the energy acceptor (PTB7-Th and NOE10) according to the Förster resonant energy transfer (FRET) theory, suggesting that FRET was realized from PBDT-TAZ to PTB7-Th and NOE10 (Huang et al., 2013). We further confirmed the existence of FRET of PBDT-TAZ and PTB7-Th through a PL experiment comparing pure PBDT-TAZ and PTB7-Th film with PBDT-TAZ:PTB7-Th blended film. When excited at 500 nm, the PBDT-TAZ:PTB7-Th blend exhibits a clearly higher PL peak intensity at 755 nm compared to the pure PTB7-Th film, while PBDT-TAZ's PL peak at 675 nm completely disappears in the blend (Figure 4C). In contrast, when excited at 700 nm, the PTB7-Th and PBDT-TAZ:PTB7-Th blended films exhibit similar PL spectra (Figure S2B). The PL responses evident at two different excitation wavelengths demonstrate that a FRET process occurs from PBDT-TAZ to PTB7-Th. It should be noted that there is competition between the energy transfer from PBDT-TAZ to PTB7-Th and the charge transfer from PBDT-TAZ to NOE10 in the BHJ ternary blends. As reported for several ternary solar cells, the energy and charge transfer processes often exhibit concurrency and intertwining (Lu et al., 2015a; Li et al., 2017a).

**Figure 5:**
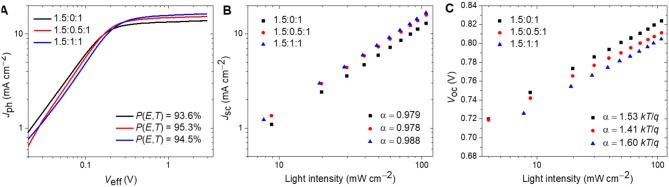
**(A)** *J*_ph_-*V*_eff_ curves of the devices; **(B)** *J*_sc_-ln(*P*_light_) properties of the devices; **(C)** *V*_oc_-ln(*P*_light_) properties of the devices.

We further studied the exciton dissociation probability *P*(*E, T*) of the all-PSCs (Koster et al., 2005). Figure 5A exhibits the photocurrent density (*J*_ph_) vs. the effective voltage (*V*_eff_) of the all-PSCs. The *P*(*E, T*) is defined by normalizing *J*_ph_ with the saturation photocurrent density (*J*_sat_) (Koster et al., 2005). Under the short-circuit conditions, the all-PSCs with ternary ratios of 1.5:0:1, 1.5:0.5:1, and 1.5:1:1 show *P*(*E, T*) values of 93.6, 95.3, and 94.5%, respectively. The all-PSC with a ternary ratio of 1.5:0.5:1 exhibits the highest *P*(*E, T*) value, further signifying that the inclusion of PTB7-Th as the third component promotes exciton dissociation in ternary devices, which is in agreement with the corresponding *J*_sc_ and EQE spectra.

Device photovoltaic properties, especially *J*_sc_ and FF values, can also be greatly impacted on charge transport properties. The mobilities of the three different ternary-blended film ratios was tested and shown in Figure S3. The results are listed in Table S2. The devices with ternary ratios of 1.5:0:1, 1.5:0.5:1, and 1.5:1:1 show electron mobilities (μ_e_) of 3.6 × 10^−4^, 3.3 × 10^−4^, and 2.4 × 10^−4^ cm^2^ V^−1^ s^−1^, respectively. The hole mobilities (μ_h_) of these blended films are 1.9 × 10^−4^, 3.2 × 10^−4^, and 4.3 × 10^−4^ cm^2^ V^−1^ s^−1^, respectively. Correspondingly, the μ_e_/μ_h_ ratios for blended films with ternary ratios of 1.5:0:1, 1.5:0.5:1, and 1.5:1:1 are 1.9, 1.0, and 0.6, respectively. The PBDT-TAZ:PTB7-Th:NOE10 blend with a ternary ratio of 1.5:0.5:1 offers optimally balanced electron/hole transport along with high FF (0.70) and *J*_sc_ (15.1 mA cm^−2^) in all-PSCs.

Charge recombination mechanisms of the devices were investigated through measurements of the light intensity dependence of the *J*_sc_ and *V*_oc_ values. The correlation of *J*_sc_ and light intensity (*P*_light_) obeys the power-law *J*_sc_ ∝ *P*_light_^α^, where α is an exponential factor that should equal 1 when all charge carriers are extracted before recombination (Cowan et al., 2010). As exhibited in Figure 5B, the α values of the fitted line for all-PSCs with ternary ratios of 1.5:0:1, 1.5:0.5:1, and 1.5:1:1 are 0.979, 0.978, and 0.988, respectively, indicating the negligible bimolecular recombination in these devices. The slope of the *V*_oc_ vs. ln(*P*_light_) curve reveals the charge recombination at open circuit conditions. Trap-assisted recombination or monomolecular recombination is dominant when the slope is 2.0 *kT*/*q*, while the slope value would be equal to 1.0 *kT*/*q* when only bimolecular recombination occurs (Cowan et al., 2010). As shown in Figure 5C, the all-PSCs with ternary ratios of 1.5:0:1, 1.5:0.5:1, and 1.5:1:1 show a slope of 1.53 *kT*/*q*, 1.41 *kT*/*q*, and 1.60 *kT*/*q*, respectively. The all-PSC with a ternary ratio of 1.5:0.5:1 exhibits the lowest slope, suggesting the low trap-assisted recombination or monomolecular recombination of that ternary ratio device. These results are consistent with the exciton dissociation measurements, charge transport analysis, and devices photovoltaic properties.

### Morphology

**Figure 6:**
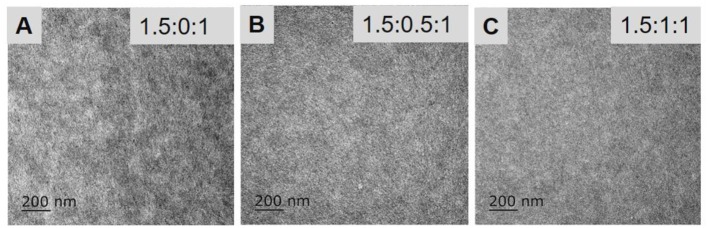
TEM images of the blend films with PBDT-TAZ:PTB7-Th:NOE10 ternary ratios of 1.5:0:1 **(A)**, 1.5:0.5:1 **(B)**, and 1.5:1:1 **(C)**.

The morphology of the BHJ blend films was tested using transmission electron microscopy (TEM). The TEM images of the blended films with three different PTB7-Th polymer-content ratios are shown in Figure 6. The blended films with ternary ratios of 1.5:0:1 and 1.5:0.5:1 exhibit similarly aligned fibrillar structures which improve charge separation and transport. Meanwhile, the near-uniform film of the 1.5:1:1 blend reveals an intimately mixed nanostructure without noteworthy phase aggregation and separation. With such morphology, charge separation and transport in the 1.5:1:1 blend are impeded, resulting relatively lower *J*_sc_ and FF in solar cells. These morphologies may be associated with the weak crystallinity of PTB7-Th; thus, the excessive loading of PTB7-Th may obstruct BHJ morphology. Overall, the microstructural morphologies of the blended films are consistent with the *J*_sc_ and FF variations of the corresponding all-PSCs, and the *J*_sc_ values can be improved and the FF values maintained when the PBDT-TAZ:PTB7-Th:NOE10 ternary ratio is approximately 1.5:0.5:1.

### Device Stability

**Figure 7:**
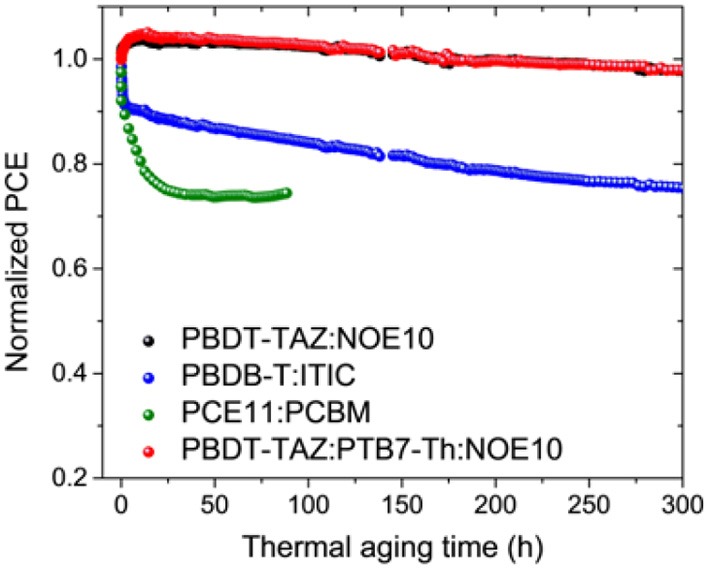
Normalized device PCE based on PBDT-TAZ:PTB7-Th:NOE10, PBDT-TAZ:NOE10, PBDB-T:ITIC, and PCE11:PCBM over the 65°C thermal aging time.

In our previous work, we demonstrated the excellent long-term storage capacity and thermal stability of the PBDT-TAZ:NOE10-based binary all-PSC system (Liu et al., 2018b). Herein, we further investigated the device stability of a PBDT-TAZ:PTB7-Th:NOE10 (1.5:0.5:1)-based ternary all-PSC under continuous thermal aging and compared it with the stability of a binary all-PSC (PBDT-TAZ:NOE10) and other two highly efficient solar cells [PCE11:PCBM (Liu et al., 2014) and PBDB-T:ITIC (Zhao et al., 2016)]. The normalized performances of the devices under 65°C thermal aging are exhibited in Figure 7, while detailed device photovoltaic properties (*V*_oc_, *J*_sc_, and FF) are depicted in Figure S4. After 300 h of continuous thermal aging at 65°C, the ternary all-PSC (PBDT-TAZ:PTB7-Th:NOE10) device holds 98% initial PCE without burn-in efficiency loss. However, the PCE11:PCBM and PBDB-T:ITIC devices exhibit obvious burn-in efficiency losses within 10–20 h of thermal aging, and these devices exhibit markedly lower long-term stabilities, including <80% initial PCE retention for PBDB-T:ITIC devices after 300 h of aging and ≈70% initial PCE retention for PCE11:PCBM devices after 25 h of aging, which could be attributed to the instability of their BHJ microstructure morphology (Li N. et al., 2017; Du et al., 2019; Zhang et al., 2019a). As in the PBDT-TAZ:NOE10 binary devices, the burn-in-free feature of the ternary devices can be attributed to the stable blend morphology (Li N. et al., 2017). All-PSCs based on NOE10 polymer acceptors, including both binary and ternary systems, show excellent long-term thermal stability. This demonstrates that NOE10 shows significant promise as an electron acceptor for practical applications in the field of PSCs.

## Conclusion

In conclusion, we have demonstrated an efficient approach to ternary all-PSCs construction by incorporating a state-of-the-art narrow-bandgap polymer, PTB7-Th, as the third component within a PBDT-TAZ:NOE10 binary system. The ternary all-PSCs achieve 8.5% PCEs within broad PTB7-Th-content ratios, representing an 18% improvement over binary all-PSCs. Compared to the binary system, the improved photovoltaic performance of ternary all-PSCs reflect the combined strengths of enhanced photon absorption, increased free charges generated through simultaneous charge and energy transfer, and balanced charge transport. Moreover, like the binary system, the ternary all-PSCs also show excellent thermal stability, maintaining 98% of their initial PCE after aging for 300 h at 65°C. This work demonstrates that the introduction of PTB7-Th as the third photoactive component in ternary PBDT-TAZ:PTB7-Th:NOE10 all-PSC construction is an effective strategy for realizing highly efficient and stable all-PSCs. It also suggests the strong potential of NOE10 as an acceptor polymer for future large-scale technological applications in both binary and ternary all-PSCs.

## Data Availability Statement

All datasets generated for this study are included in the article/Supplementary Material.

## Author Contributions

XL conceived the idea, synthesized and characterized the polymer acceptor, performed the fabrication of solar cells and data analysis, and collected TEM images. CZ performed the thermal stability experiments of the devices supervised by NL and CB. SP performed the SCLC experiments. XL and CD prepared the manuscript. All authors commented on the manuscript. CD, FH, and YC supervised the project.

## Conflict of Interest

The authors declare that the research was conducted in the absence of any commercial or financial relationships that could be construed as a potential conflict of interest.
