# Supplementary material for: Ternary All-Polymer Solar Cells With 8.5% Power Conversion Efficiency and Excellent Thermal Stability
Source: Front Chem. 2020 Apr 21;8:302. doi: 10.3389/fchem.2020.00302 (PMC7212455; doi:10.3389/fchem.2020.00302)
Supplement: Supplementary file 1 [file Data_Sheet_1.PDF]

# *Supplementary Material*

# *Ternary All-Polymer Solar Cells with 8.5% Power Conversion Efficiency and Excellent Thermal Stability*

**Xi Liu[1,2*], Chaohong Zhang[3,4], Shuting Pang[2], Ning Li[3,5], Christoph J. Brabec[3,5], Chunhui Duan[2*], Fei Huang[2*], Yong Cao[2]**

[1]School of Textile Materials and Engineering, Wuyi University, Jiangmen 529020, P. R. China

[2]State Key Laboratory of Luminescent Materials and Devices, Institute of Polymer Optoelectronic Materials and Devices, South China University of Technology, Guangzhou 510640, P. R. China

[3]Institute of Materials for Electronics and Energy Technology (i-MEET), Friedrich-Alexander-Universität Erlangen-Nürnberg, Martensstrasse 7, 91058 Erlangen, Germany

[4]SUSTech Academy for Advanced Interdisciplinary Studies, Southern University of Science and Technology, Shenzhen 518055, P. R. China

[5]Helmholtz Institute Erlangen-Nürnberg for Renewable Energy (HI ERN), Immerwahrstrasse 2, 91058 Erlangen, Germany

*** Correspondence:**
Xi Liu
liuxi_wyu@163.com

Chunhui Duan
duanchunhui@scut.edu.cn

Fei Huang
msfhuang@scut.edu.cn

## Experimental Section

**General Details.** UV-vis spectra were recorded on a Shimadzu UV-3600 spectrophotometer. Cyclic voltammetry data were measured on a CHI600D electrochemical workstation by using $Bu_4NPF_6$ (0.1 M) in acetonitrile as electrolyte and glassy-carbon, platinum, and saturated calomel electrode as the working, counter, and reference electrode, respectively. Potentials were referenced to the ferrocenium/ferrocene couple by using ferrocene as an internal standard.

**Fabrication and characterization of solar cells.** The all-polymer solar cells (all-PSCs) with a conventional device structure of ITO/PEDOT:PSS/BHJ active layer/PFN-Br/Ag were fabricated. First, PEDOT:PSS (CLEVIOS™ P VP AI 4083) was spin-coated on top of a cleaned ITO and annealed in air at 150 °C for 15 min to form ~40 nm layer. Subsequently, the active layer with an optimal thickness of ca. 105 nm was formed by spin-coating the mixed solution of PBDT-TAZ:PTB7-Th:NOE10 (with different ternary ratios) in chloroform (with 2% diphenyl ether, v/v) on top of the PEDOT:PSS layer. After that, the active layers were annealed at 160 °C for 5 min. After spin-coating of 5 nm PFN-Br as cathode interface, a 100 nm Ag layer were sequentially deposited by thermal evaporation through a shadow mask in a vacuum chamber at a pressure of $4\times10^{-7}$ Torr. The active layer area of the device was defined to be 0.0516 $cm^2$, which was further confined as 0.04 $cm^2$ by a non-refractive mask to improve the accuracy of measurements. The photovoltaic performance was measured under an AM 1.5G solar simulator (Taiwan, Enlitech SS-F5). The current density-voltage (*J–V*) characteristics were recorded with a Keithley 2400 source meter. The light intensity was 100 mW $cm^{-2}$ as calibrated by a China general certification center (CGC) certified reference monocrystal silicon cell (Enlitech). The external quantum efficiency (EQE) spectra were performed on a commercial EQE measurement system (Taiwan, Enlitech, QE-R3011). The light intensity at each wavelength was calibrated by a standard single-crystal Si photovoltaic cell.

**Fabrication and characterization of SCLC devices.** Devices were fabricated to measure hole and electron mobility by using the space-charge-limited current (SCLC) method. The device structures of the hole-only devices and electron-only are ITO/PEDOT:PSS/blend films/$MoO_3$/Ag and ITO/ZnO/blend films/PFN-Br/Ag, respectively. The mobility was determined by fitting the dark current to the model of a single-carrier SCLC model, which is described by the equation $J$ =

$(9/8)\varepsilon_0\varepsilon_r\mu((V^2)/(d^3))$, where $J$ is the current, $\mu$ is the zero-field mobility, $\varepsilon_0$ is the permittivity of free space, $\varepsilon_r$ is the relative permittivity of the material, $d$ is the thickness of the active layers, and $V$ is the effective voltage. The effective voltage was obtained by subtracting the built-in voltage ($V_{bi}$) and the voltage drop ($V_s$) from the series resistance of the whole device except for the active layers from the applied voltage ($V_{appl}$), $V = V_{appl} - V_{bi} - V_s$. The charge carrier mobility was calculated from the slope of the $J^{1/2}$~$V$ curves.

**PL quenching experiments.** The PL quenching experiments were recorded by Shimadzu RF-6000 spectrometer @ different excitation wavelengths for the corresponding films.

**Transmission electron microscopy (TEM).** Transmission electron microscopy was performed on a Tecnai G2 Sphera transmission electron microscope (FEI) operated at 200 kV.

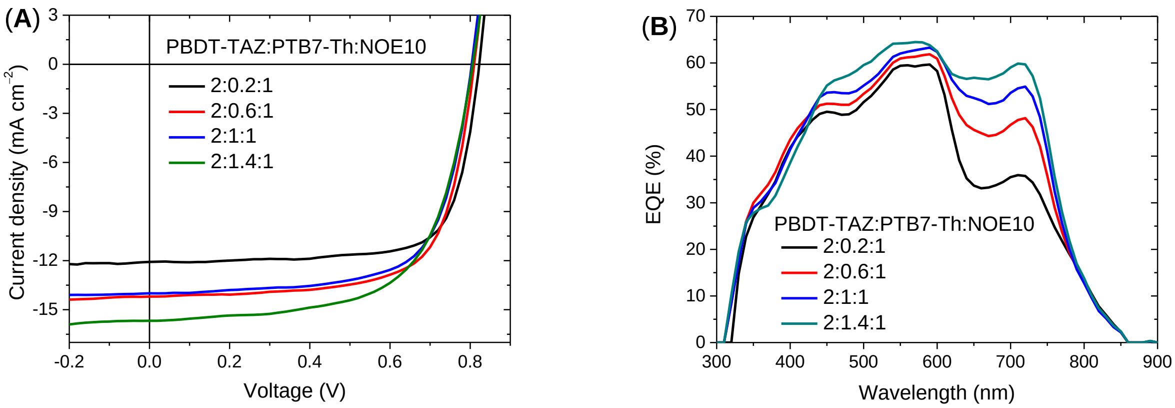


**Figure S1.** (**A**) Current density–voltage characteristics of the all-PSCs under AM1.5G illumination at 100 mW cm$^{-2}$; (**B**) EQE spectra of the corresponding all-PSC devices.

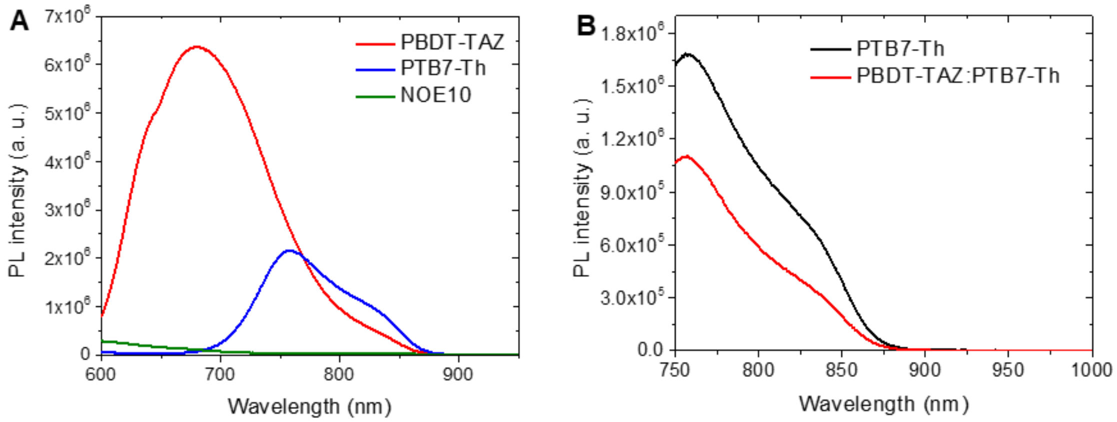


**Figure S2.** (A) PL spectra of the neat polymers excited at 500 nm; (B) PL spectra of neat PTB7-Th, and PBDT-TAZ:PTB7-Th blend film excited at 700 nm.

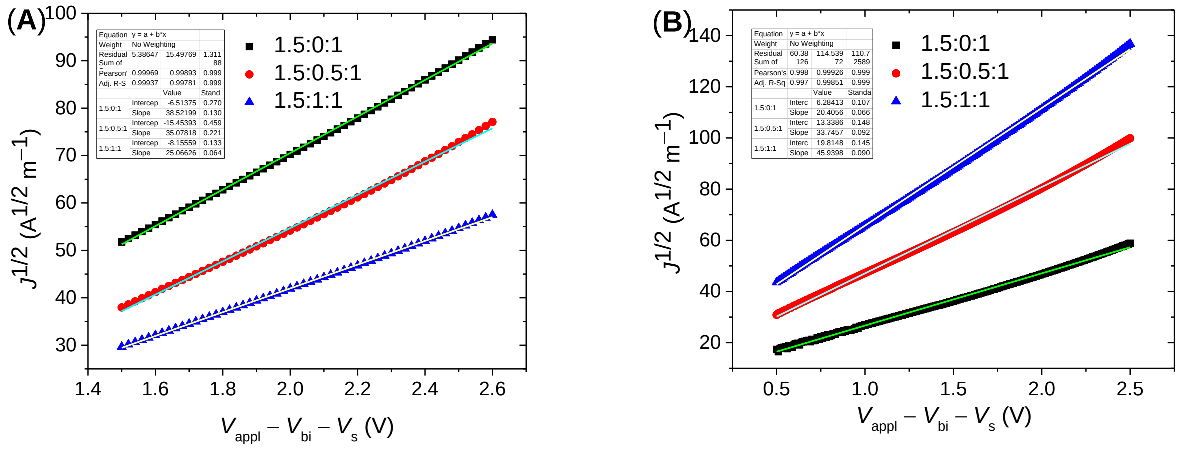


**Figure S3.** The $J^{1/2}$ *versus* $V_{\text{appl}} - V_{\text{bi}} - V_{\text{s}}$ characteristic in SCLC regions of electron-only devices (**A**) and hole-only devices (**B**) for the corresponding ternary ratio-based blend films.

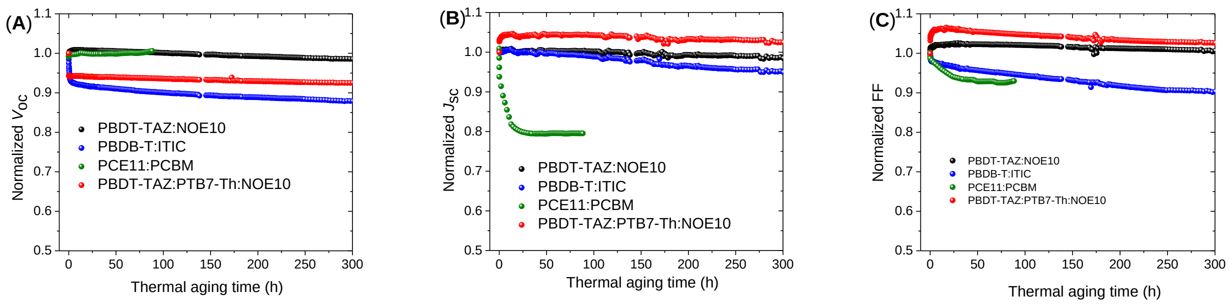

**Figure S4.** The normalized device parameters of the solar cells of PBDT-TAZ:NOE10, PBDB-T:ITIC, PCE11:PCBM, and PBDT-TAZ:PTB7-Th:NOE10 over the 65 °C thermal aging time. (**A**) $V_{oc}$, (**B**) $J_{sc}$, and (**C**) FF.

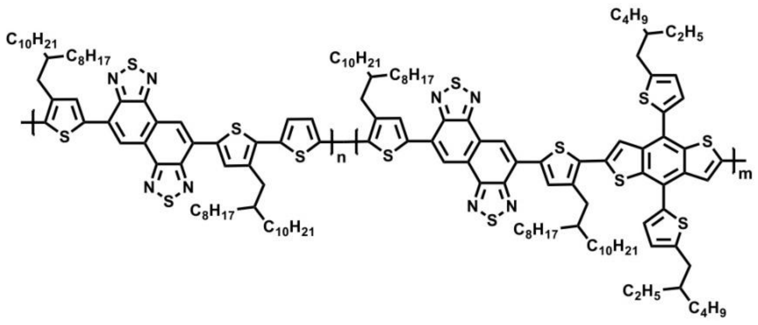

**Figure S5.** Chemical structure of PNT-X.

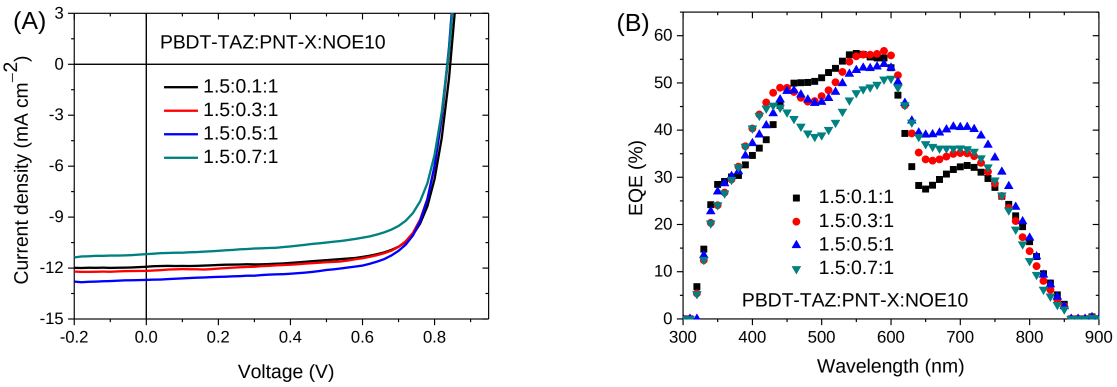


**Figure S6.** (**A**) Current density–voltage characteristics of the all-PSCs based on PBDT-TAZ:PNT-X:NOE10; (**B**) EQE spectra of the corresponding all-PSC devices.

**Table S1** Photovoltaic properties of the devices under AM1.5G illumination at 100 mW cm$^{-2}$.

| D1:D2:A[a] | $V_{oc}$ [V] | $J_{sc}$ [mA cm$^{-2}$] | FF | PCE [%][b] |
|---|---|---|---|---|
| 2:0.2:1 | 0.823 | 12.1 | 0.75 | 7.4 (7.4 ± 0.2) |
| 2:0.6:1 | 0.810 | 14.2 | 0.70 | 8.0 (8.0 ± 0.1) |
| 2:1:1 | 0.804 | 14.0 | 0.69 | 7.7 (7.6 ± 0.1) |
| 2:1.4:1 | 0.806 | 15.7 | 0.64 | 8.1 (8.0 ± 0.1) |

[a]D1 (PBDT-TAZ), D2 (PTB7-Th), A (NOE10)

**Table S2** Electron and hole mobilities obtained by SCLC method.

| PBDT-TAZ:PTB7-Th:NOE10 | $\mu_e$ [cm$^2$ V$^{-1}$ s$^{-1}$] | $\mu_h$ [cm$^2$ V$^{-1}$ s$^{-1}$] | $\mu_e/\mu_h$ |
|---|---|---|---|
| 1.5:0:1 | $3.6 \times 10^{-4}$ | $1.9 \times 10^{-4}$ | 1.9 |
| 1.5:0.5:1 | $3.3 \times 10^{-4}$ | $3.2 \times 10^{-4}$ | 1.0 |
| 1.5:1:1 | $2.4 \times 10^{-4}$ | $4.3 \times 10^{-4}$ | 0.6 |

**Table S3** Photovoltaic properties of the devices under AM1.5G illumination at 100 mW cm$^{-2}$.

| D1:D2:A[a] | $V_{oc}$ [V] | $J_{sc}$ [mA cm$^{-2}$] | FF | PCE [%][b] |
|---|---|---|---|---|
| 1.5:0.1:1 | 0.843 | 11.9 | 0.75 | 7.5 (7.4 ± 0.2) |
| 1.5:0.3:1 | 0.836 | 12.2 | 0.74 | 7.5 (7.5 ± 0.1) |
| 1.5:0.5:1 | 0.835 | 12.7 | 0.73 | 7.7 (7.6 ± 0.1) |
| 1.5:0.7:1 | 0.836 | 11.2 | 0.71 | 6.7 (6.7 ± 0.1) |

[a]D1 (PBDT-TAZ), D2 (PNT-X), A (NOE10)
